# Myeloid sarcomas: a histologic, immunohistochemical, and cytogenetic study

**DOI:** 10.1186/1746-1596-2-42

**Published:** 2007-10-31

**Authors:** Borislav A Alexiev, Wenle Wang, Yi Ning, Saranya Chumsri, Ivana Gojo, William H Rodgers, Sanford A Stass, Xianfeng F Zhao

**Affiliations:** 1 Department of Pathology, University of Maryland Medical Center, 22 S Greene Street, Baltimore, MD, USA, 21201; 2 Department of Internal Medicine, Division Hematology Oncology, University of Maryland Medical Center, Marlene and Stewart Greenebaum Cancer Center, 22 S. Greene Street, Baltimore, MD, USA, 21201

## Abstract

**Context. -:**

Myeloid sarcoma (MS) is a neoplasm of immature granulocytes, monocytes, or both involving any extramedullary site. The correct diagnosis of MS is important for adequate therapy, which is often delayed because of a high misdiagnosis rate.

**Objective. -:**

To evaluate the lineage differentiation of neoplastic cells in MS by immunohistochemistry, and to correlate the results with clinicopathologic findings and cytogenetic studies.

**Design. -:**

Histologic and immunohistochemical examinations were performed on formalin-fixed paraffin-embedded tissue samples from 13 cases of MS. They were classified according to the World Health Organization criteria. Chromosomal analysis data were available in 11 cases. Clinical, pathological, and cytogenetic findings were analyzed.

**Results. -:**

The study included six male and seven female patients with an age range of 25 to 72 years (mean, 49.3 years) and a male to female ratio of 1:1.2. MS de novo occurred in 4/13 (31%) of cases examined. The most sensitive immunohistochemical markers were CD43 and lysozyme present in all cases with MS (13/13, 100%). All de novo MS showed a normal karyotype, monoblastic differentiation, and lack of CD34. The most common chromosomal abnormalities in MS associated with a hematopoietic disorder were trisomy 8 and inv(16) (2/11, 18%).

**Conclusion. -:**

An immunohistochemical panel including CD43, lysozyme, myeloperoxidase (MPO), CD68 (or CD163), CD117, CD3 and CD20 can successfully identify the vast majority of MS variants in formalin-fixed paraffin-embedded tissue sections. The present report expands the spectrum of our knowledge showing that de novo MS has frequent monoblastic differentiation and frequently carries a normal karyotype.

## Background

Myeloid sarcoma (MS) is a tumor mass of myeloblasts or immature myeloid cells occurring in an extramedullary site or in the bone [1]. The tumor can involve any part of the body, but commonly involved sites include subperiosteal bone structures of the skull, paranasal sinuses, sternum, ribs, vertebrae, and pelvis; lymph nodes and skin are also common sites [1]. MS may occur de novo or concurrently with acute myeloid leukemia (AML) or a myeloproliferative disorder [1]. The rate of occurrence is approximately 1.4% to 9% of patients with AML [2,3]. MS is frequently mistaken for non-Hodgkin lymphoma (NHL), small round cell tumor (neuroblastoma, rhabdomyosarcoma, Ewing sarcoma/PNET, and medulloblastoma), and undifferentiated carcinoma. The diagnosis is missed in about 50% of cases when immunohistochemistry is not used [4]. The most common suggested diagnosis was that of a NHL [5].

The present study was designated to evaluate the lineage differentiation of neoplastic cells in MS by immunohistochemistry, and correlate the results with clinicopathologic findings and cytogenetic studies.

## Material

Thirteen patients with a histologic diagnosis of myeloid sarcoma were included in the present study. The initial diagnosis was made on core biopsies (3 cases) and surgical specimens (10 cases). The specimens were fixed in 10% formaldehyde and embedded in paraffin. Five-micron tissue sections were stained with hematoxylin-eosin. The use of paraffin blocks for this study meets Institutional Review Board and Health Insurance Portability and Accountability Act requirements, and has been approved by the Institutional Review Board at the University of Maryland.

### Immunohistochemistry

Immunohistochemical staining was performed using an automated slide preparation system (Benchmark XT, Ventana, Tuscon, AZ), a Ventana Enhanced DAB Detection Kit (Ventana, Tucson, AZ), and commercially available prediluted monoclonal antibodies: CD163 (NeoMarkers), CD4 (Biocare Medical), myeloperoxidase, lysozyme, CD3, CD4, CD8, CD15, CD20, CD34, CD43, CD68, CD79a, CD117, Factor VIII (FVIII), and glycophorin A (all Ventana, Tucson, AZ).

### Chromosomal Study

Chromosomal analysis was performed on tumor specimens from 11 patients at diagnosis. Cells were cultured in RPMI 1640 medium with 20% fetal bovine serum for 24 and 48 hours, respectively. Metaphase cells were analyzed following standard G-banding method. Their karyotypes were interpreted according to the International System for Human Cytogenetic Nomenclature.

## Results

### Demographic data

The clinical findings are shown in Table 1. The study included six male and seven female patients with an age range of 25 to 72 years (mean, 49.3 years) and a male to female ratio of 1:1.2. MS de novo occurred in 4/13 (31%) cases examined.

**Table 1:** Clinical findings in patients with myeloid sarcoma

Case	Age, year/sex	Site	Bone marrow
1	25/M	Scrotum	Uninvolved
2	66/M	Kidney	RAEB-2*
3	27/M	Testis	AML, M2
4	54/F	Colon	AML, M2
5	58/M	Orbit	Uninvolved
6	72/M	Skin	Uninvolved
7	46/F	Lymph Node	Uninvolved
8	62/F	Gingiva	Myelofibrosis
9	36/F	Vagina	AML, M1
10	38/M	T5, T7	CML/BP**
11	51/F	Gallbladder	AML, M5b
12	40/F	Breast	AML, M1
13	38/F	Breast	AML, M4

*RAEB-2: Refractory anemia with excess blasts 2

**CML/BP: Chronic myelogeneous leukemia, blast phase

### Sites of involvement

MS occurred in a variety of extramedullary sites (Table 1). Nine of thirteen cases with MS (69%) had a synchronous involvement of the bone marrow by acute leukemia, myeloproliferative, or myelodysplastic disorder.

### Histology and immunohistochemistry

The pathologic diagnoses are shown in Table 2.

**Table 2:** Pathologic diagnosis in patients with myeloid sarcomas

Case	Pathologic diagnosis
1	Monoblastic sarcoma*
2	Myelomonocytic sarcoma
3	Granulocytic sarcoma, differentiated
4	Granulocytic sarcoma, differentiated
5	Monoblastic sarcoma*
6	Monoblastic sarcoma*
7	Monoblastic sarcoma*
8	Myelomonocytic sarcoma
9	Granulocytic sarcoma, immature
10	Granulocytic sarcoma, differentiated
11	Monocytic sarcoma
12	Granulocytic sarcoma, immature
13	Myelomonocytic sarcoma

* MS de novo.

Immature granulocytic sarcomas (IGS) were characterized by the presence of numerous (> 90%) blasts with high N/C ratio, round or oval nucleus, dispersed chromatin and prominent nucleolus. The cytoplasm of the majority of neoplastic cells was agranular with a varying degree of basophilia. Eosinophilic granulation was notable in a minority of a cell population. The neoplastic cells in IGS showed reactivity with CD34, CD43, CD117, and lysozyme. MPO was present in a variable number of blasts, but always <10%. Focal weak reactivity of the neoplastic cells with antibodies to CD68 and/or CD163 was also noted. Differentiated granulocytic sarcomas (DGS) showed maturation to more mature neutrophils (>10% of neoplastic cells). The neoplastic cells in DGS showed strong reactivity with CD43, MPO, CD15, lysozyme, and variably expressed CD117. CD68 and CD163 were positive in <20% of neoplastic cells.

**Figure 1:**
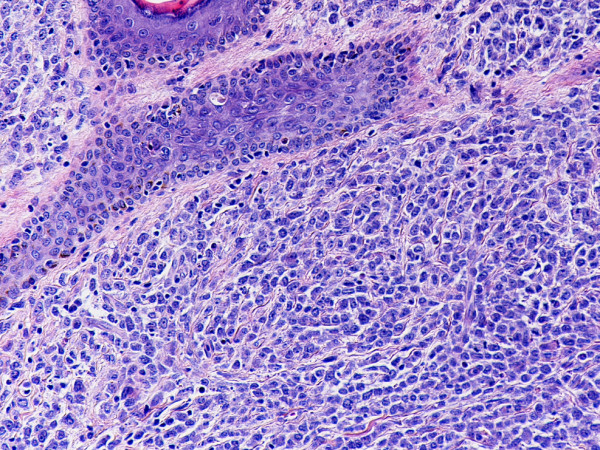
Monoblastic sarcoma de novo, skin. (hematoxylin-eosin, × 100).

**Figure 2:**
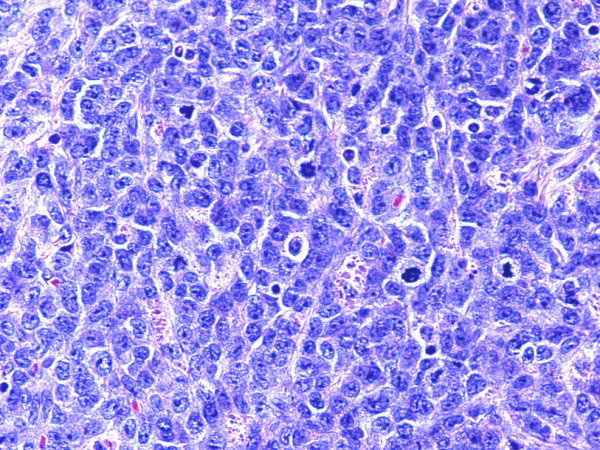
Monoblatic sarcoma de novo, skin. Multiple mitotic figures are seen. (hematoxylin-eosin, × 400).

**Figure 3:**
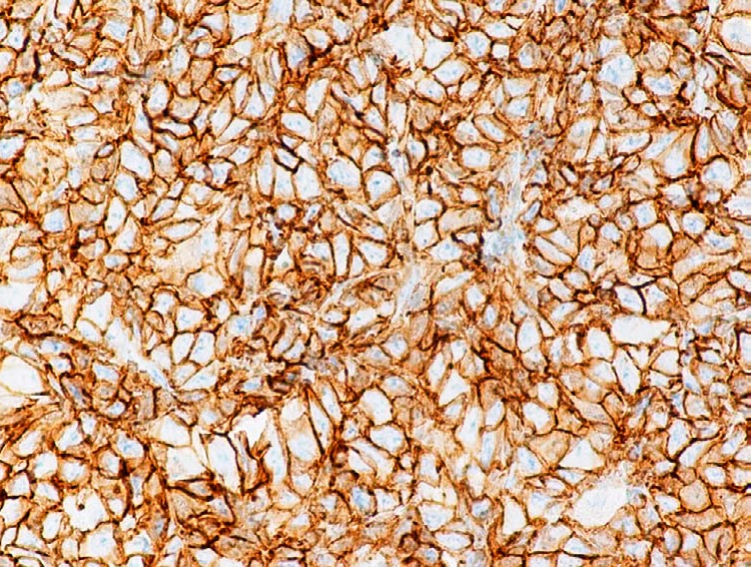
Monoblastic sarcoma de novo. Neoplastic cells are positive for CD43. (B-SA, anti-CD43, × 400).

**Figure 4:**
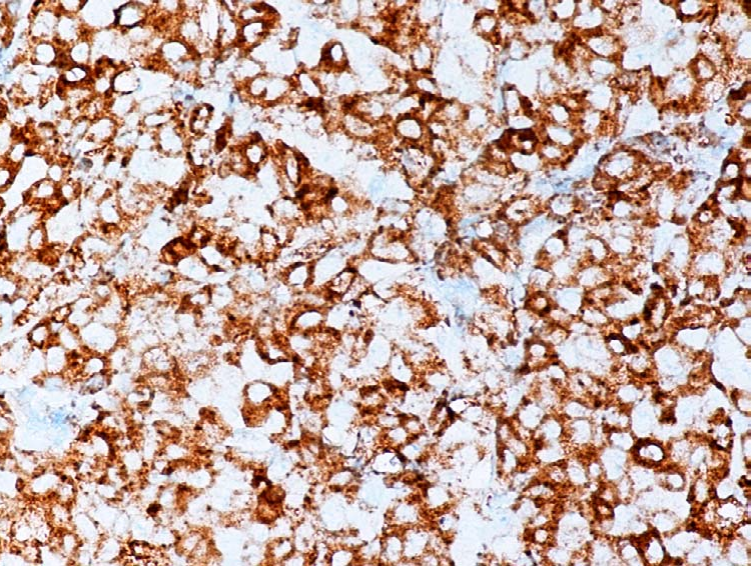
Monoblastic sarcoma de novo. Neoplastic cells are positive for CD163. (B-SA, anti-CD163, × 400).

**Figure 5:**
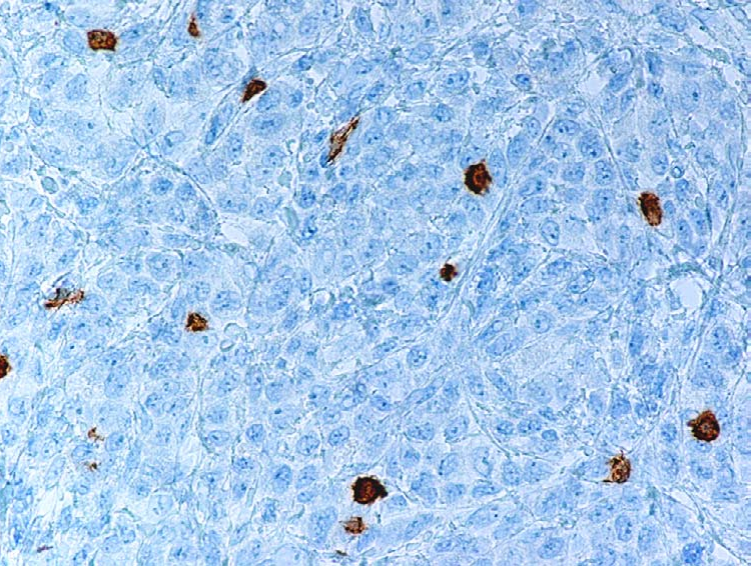
Monoblastic sarcoma de novo. Neoplastic cells are negative for CD3. Residual T-lymphocytes are positive with CD3 antibody. (B-SA, anti-CD3, × 400).

**Figure 6:**
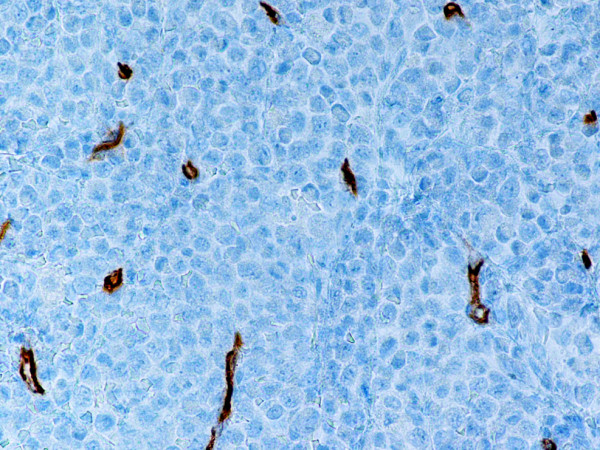
Monoblastic sarcoma de novo. One single neoplastic cell is positive for MPO. (B-SA, anti-MPO, × 400).

**Figure 7:**
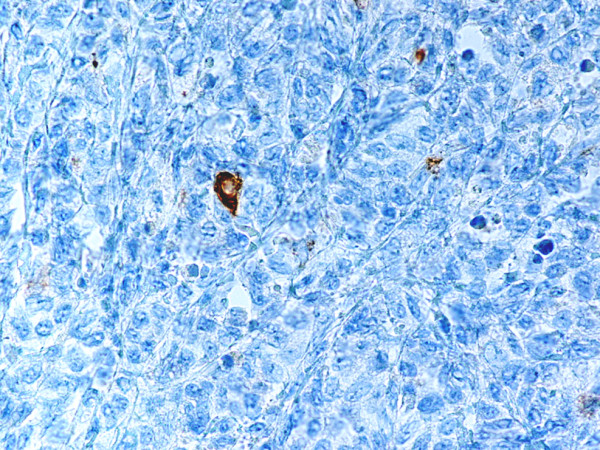
Monoblastic sarcoma de novo. Neoplastic cell are negative for CD34. Stain for CD34 reveals endothelial cells. (B-SA, anti-CD34, × 400).

Monoblastic sarcomas (MBLS) were composed of a large population (> 80%) of monoblasts. The neoplastic cells were large, with abundant eosinophilic cytoplasm, round or oval nuclei with dispersed chromatin and one or more prominent nucleoli. Promonocytes showed more irregular, delicately convoluted nuclear features. MBLS were strongly positive for CD43, lysozyme, CD68, CD163, weakly for CD4, and negative for CD34. Scattered MPO positive cells were also noted (Figs. 1, 2, 3, 4, 5, 6, 7).

**Figure 8:**
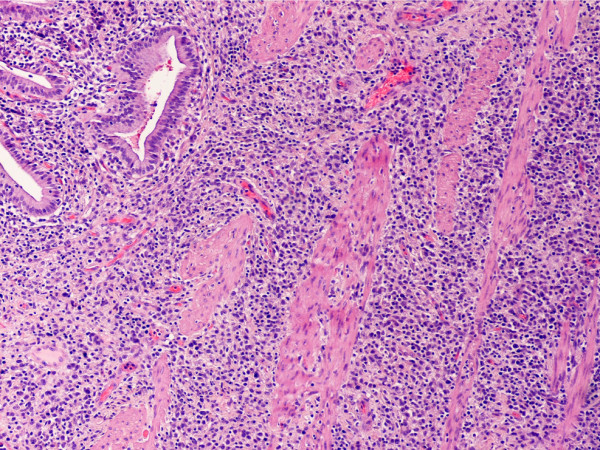
Monocytic sarcoma, gallbladder. (hematoxylin-eosin, × 100).

**Figure 9:**
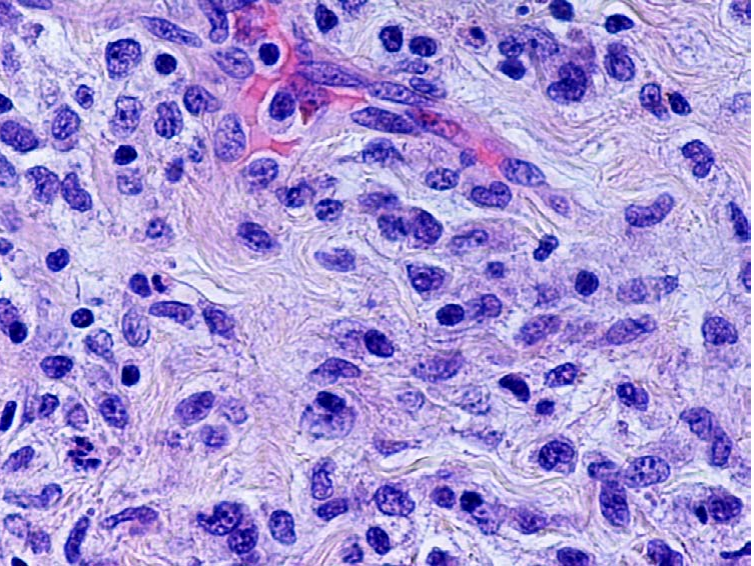
Monocytic sarcoma, gallbladder. Neoplastic cells have very dispersed chromatin and inconspicuous nucleoli. Note irregular and delicately convoluted nuclear configuration. (hematoxylin-eosin, × 400).

**Figure 10:**
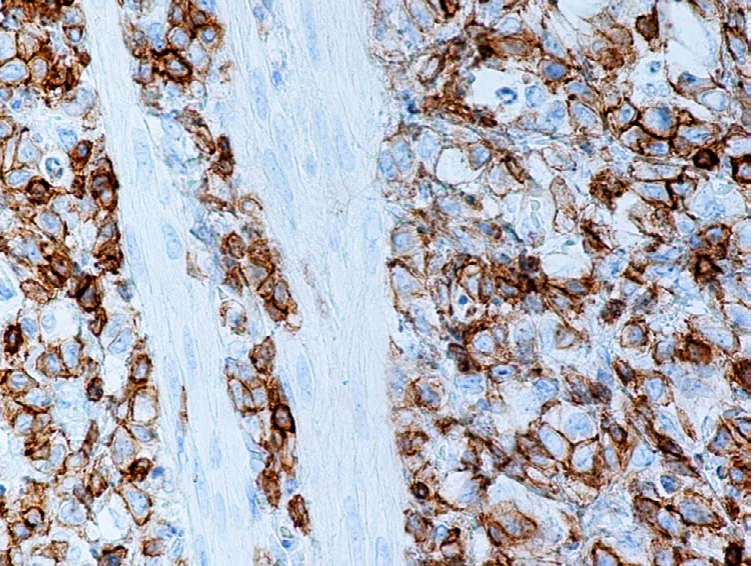
Monocytic sarcoma, gallbladder. Neoplastic cells are positive for CD43. (B-SA, anti-CD43, × 400).

**Figure 11:**
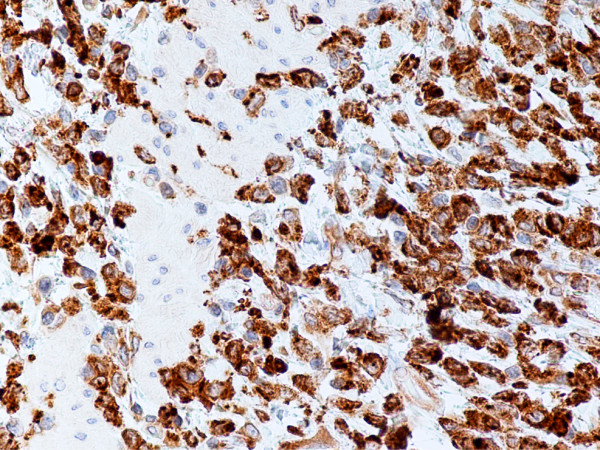
Monocytic sarcoma, gallbladder. Neoplastic cells are positive for CD68. (B-SA, anti-CD68, × 400).

In monocytic sarcoma (MCS), the majority of neoplastic cells showed marked nuclear lobulation and stained strongly with antibodies to CD43, lysozyme, CD68, CD163, and variably with MPO (Figs. 8, 9, 10, 11).

Myelomonocytic sarcomas (MMS) were characterized by proliferation of both neutrophilic and monocytic precursors with above described morphologic and immunohistochemical features each comprising >20% of neoplastic cells. The cases were notable for increased numbers of eosinophils containing large eosinophilic granules in the cytoplasm.

The neoplastic cells in all MS examined did not react with antibodies to FVIII, and glycophorin A. T and B lymphocyte lineage-specific antigens such as CD3, CD20, and CD79a were typically absent.

All MS were morphologically and immunophenotypically analogous to their leukemic counterparts.

### Chromosomal study

**Figure 12:**
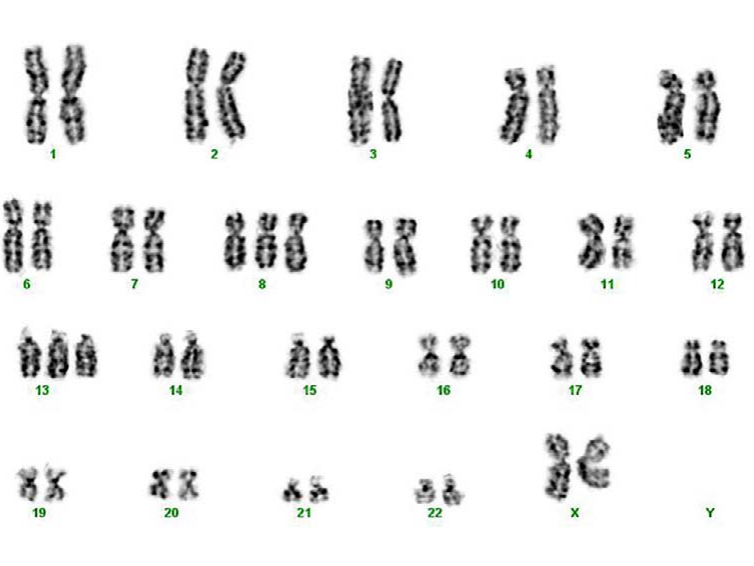
Monocytic sarcoma, gallbladder. Chromosomal analysis reveals trisomy 8 and trisomy 13. (standard G-banding)

The cytogenetic findings are shown in Table 3. Chromosomal abnormalities were observed in 6/11 (55%) cases examined. All patients with MS occurring de novo had a normal karyotype (4/4, 100%). The most common genetic abnormalities were the presence of an extra chromosome 8 and inv(16) (2/11, 18%) (Fig. 12).

**Table 3:** Cytogenetic findings in patients with myeloid sarcoma

Case	Cytogenetics	Pathologic diagnosis
1	46, XY	Monoblastic sarcoma*
2	47, XY, +21	Myelomonocytic sarcoma
3	46, XY, del(8)(q24.2)	DGS***
4	N/A	DGS***
5	46, XX	Monoblastic sarcoma*
6	46, XY	Monoblastic sarcoma*
7	46, XX	Monoblastic sarcoma*
8	N/A	Myelomonocytic sarcoma
9	46, XX	IGS**
10	47, XY, +8, t(9;22)(q34;q11.2)	DGS***
11	48, XX, +8, +13	Monocytic sarcoma
12	47, XX, t(4;7)(p12;p11.2), Inv(9)(p12q13), inv(16)(p13.1q22)	IGS**
13	46, XX, inv (16)(p13.1q22)	Myelomonocytic sarcoma

* MS de novo

** IGS: Immature granulocytic sarcoma

***DGS: Differentiated granulocytic sarcoma

## Comments

MS is a neoplasm of immature granulocytes, monocytes, or both involving any extramedullary site [6]. The neoplasm usually occurs in patients with acute myeloid leukemia, myelodysplastic or myeloproliferative disorder. It may rarely precede peripheral blood or bone marrow involvement, presenting a diagnostic challenge. In our study, all patients were adults with a slight female predominance. In agreement with previous reports, the neoplasms occurred in different locations including the orbit, gingiva, skin, breast, vertebral bodies, GI tract, and genito-urinary system. [6-16].

With extensive morphological and immunohistochemical analyses, the MS were classified into five types: a) immature granulocytic sarcoma (IGS); b) differentiated granulocytic sarcoma (DGC); c) monoblastic sarcoma (MBLS); d) monocytic sarcoma (MS), and myelomonocytic sarcoma (MMS) (Table 2). These tumors were also morphologically and immunophenotypically analogous to their leukemic counterparts. In agreement with previous reports [5], CD43 and lysozyme were the most sensitive markers staining a large proportion of neoplastic cells in all tumors examined (13/13, 100%). MPO and CD117 were the most sensitive of the markers for myeloid differentiation while monocytic precursors consistently strongly expressed CD68 and CD163. Differential diagnosis of MS includes several entities, the majority of which can be readily distinguished using a combination of morphologic and immunohistochemical evaluation. MS is frequently mistaken for malignant B-cell or T-cell lymphoma, especially when it presents without leukemic manifestation [3]. An incomplete workup may be misleading because NHL and MS share morphologic similarities and both express some leukocyte antigens, such as CD43 and CD45. In our study, B-cell and T-cell lymphomas were excluded by negative stains for CD20, CD79a and CD3, respectively. In addition, blastic NK cell lymphoma can mimic IGS, or MBLS. The neoplastic cells consistently express CD43, CD4, CD56, and, unlike MS, are negative for MPO, CD33, CD117, lysozyme, and CD68. Burkitt lymphoma could be excluded by negative immunoreactivity for B-cell-associated antigens, a relatively low proliferation rate, and lack of t(8;14) or t(2;8), and t(8;22) translocations.

The findings are in agreement with previous observations and suggest that an immunohistochemical panel including CD43, MPO, CD117, CD68 (or CD163), CD3 and CD20 can successfully identify the majority of MS in formalin-fixed, paraffin-embedded tissue specimens [17-22]. Using the antibody panel mentioned above, we reached the correct diagnosis in all cases examined. In cases with megakaryoblastic and/or erythroblastic differentiation, diagnosis can be confirmed by inclusion of one or more lineage-specific markers, such as FVIII, CD41, CD61, glycophorin A, and hemoglobin A in the diagnostic panel [1,23].

MS has been described in association with a variety of chromosomal abnormalities [1,24]. In particular, t(8;21)(q22;q22) and inv(16) are regarded as recurrent aberrations in GS [1,3,9,15] while translocations involving 11q23 have been detected in MBLS [1]. In this study, chromosomal aberrations were detected in 55% (6/11) of the cases. The commonest abnormalities were +8, and inv(16) occurring in 18% (2/11) of MS. DGS (n = 1) and MCS (n = 1) carried trisomy 8 (n = 1) while inv(16) was observed in IGS (n = 1) and MMS (n = 1). The findings support the recent observation of Deeb et al. [24] who described chromosome 8 abnormalities as the most common genetic aberration in MS. In this study, all cases carrying chromosomal abnormalities (6/6, 100%) had simultaneous AML, myeloproliferative disorder or MDS. Similar to what was reported by others [25], we found that inv(16) was associated with myelomonocytic differentiation in at least one case. Inv(16) was also identified in another case that was classified as IGS with multiple cytogenetic abnormalities. Furthermore, both cases involved the breast, in contrast to earlier reports of intestinal involvement by inv(16) positive MS with monocytic differentiation. All patients with de novo MS showed a normal karyotype (4/4, 100%), monoblastic differentiation, and lack of CD34. Previous studies revealed a correlation of monocytic differentiation, lack of CD34, and normal cytogenetics with nucleophosmin gene (NPM1) abnormalities [26]. In adults, NPM1 gene mutations have been identified in 50% to 60% of all AML cases with a normal karyotype [26]. Whether this newly identified molecular marker can aid in stratification of patients is under clinical investigation. In addition, a recent study showed a significant correlation between extramedullary involvement and coexpression of MCP-1/CCR2 by M4–M5 blasts which might help to explain some aspects of the pattern of invasion in MS with monocytic differentiation [27].

The correct diagnosis of MS is important for adequate therapy, which is often delayed because of a high misdiagnosis rate [7]. Previous studies have indicated that the biologic behavior is dramatic irrespective of presentation, age, sex, phenotype and cytogenetics [25]. Conversely to that reported by Pileri et al. [25], other reports showed a shorter median survival of patients with chromosome 8 abnormalities [2] and better prognosis for patients with GS [9]. From our experience, the occurrence of GS in patients with hematological abnormalities has a negative impact on median survival and prognosis (unpublished observations).

The present report expands the spectrum of our knowledge showing that de novo MS has frequent monoblastic differentiation and frequently carries a normal karyotype. The most common genetic aberrations in MS associated with hematopoietic disorders were trisomy 8 and inv(16).

## Competing interests

The author(s) declare that they have no competing interests.

## Authors' contributions

BAA evaluated the H&E and immunohistochemical stains, confirmed the diagnosis, designed the report and drafted the manuscript.

YN performed the cytogenetic studies.

SC and IG provided relevant clinical information.

WHR, SAS and XFZ provided consultation.

All authors read and approved the final manuscript.
